# Correlation between body mass index and prostate volume in benign prostatic hyperplasia patients undergoing holmium enucleation of the prostate surgery

**DOI:** 10.1186/s12894-020-00753-9

**Published:** 2021-06-10

**Authors:** Ken Batai, Michael Phung, Robert Bell, Aye Lwin, Kieran A. Hynes, Elinora Price, Karleen M. Meiklejohn, Erika R. Bracamonte, Joel T. Funk

**Affiliations:** 1 Department of Urology, The University of Arizona, 1501 N Campbell Ave, PO Box 245077, Tucson, AZ 85724-5077 USA; 2 Department of Urology, University of California Los Angeles, 10833 Le Conte Avenue, Box 951738, Los Angeles, CA 90095-1738 USA; 3 Department of Pathology and Immunology, Washington University in St. Louis, 660 S Euclid Ave, Campus, Box 8118, St. Louis, MO 63110 USA; 4 Department of Surgery, University of Mississippi Medical Center, 2500 N State St, Jackson, MS 39216 USA; 5 Department of Surgery, University of Arizona, 1501 N. Campbell Ave., Tucson, AZ 85724 USA; 6 Department of Pathology, University of Arizona, 1501 N. Campbell Ave., Tucson, AZ 85724 USA

**Keywords:** Obesity, Co-morbidity, Health disparities

## Abstract

**Background:**

Benign prostatic obstruction (BPO) due to benign prostatic hyperplasia (BPH) is a leading cause of morbidity in men over the age of 40. This study examined whether there was an association between body mass index (BMI) and pre-operative prostate volume and whether expression of two genes, alpha-2-macroglobulin (*A2M*) and transforming growth factor beta 3 (*TGFB3*), was correlated with BMI, pre-operative prostate volume, and age at surgery.

**Methods:**

Medical records of patients who underwent holmium enucleation of the prostate surgery for treatment of BPO were retrospectively reviewed. Surgical specimens were obtained from formalin-fixed paraffin-embedded blocks, and expression of the targeted genes was quantified using a real time PCR approach. Linear regression analysis was performed to assess association between BMI and prostate volume adjusting for demographic characteristics and co-morbidity. Spearman’s correlation was used to examine whether gene expression was correlated with BMI, prostate volume, and age at surgery.

**Results:**

A total of 278 patients were identified, including 62.9% European Americans (n = 175) and 27.7% Hispanic Americans (n = 77). BMI was significantly correlated with prostate volume (Spearman’s *rho* = 0.123, *P* = 0.045). In linear regression analysis, BMI was positively associated with prostate volume (*β* = 0.01, *P* = 0.004), while hyperlipidemia was negatively associated with prostate volume (*β* = −0.08, *P* = 0.02). A trend for a positive association was also observed for diabetes (*β* = 0.07, *P* = 0.099). In the race/ethnicity stratified analysis, age at surgery showed a trend for significantly positive association with prostate volume in European Americans (*β* = 0.005, *P* = 0.08), but not in Hispanic Americans. Expression of the *A2M* gene in the stroma was negatively correlated with age at surgery (*P* = 0.006). *A2M* expression in the gland was positively correlated with prostate volume among older men (Age ≥ 70, *P* = 0.01) and overweight men (BMI 25–30, *P* = 0.04). *TGFB3* expression in the gland was positively correlated with BMI (*P* = 0.007) among older men.

**Conclusions:**

This study demonstrated the positive correlation between BMI and prostate volume. Expression of *TGFB3* and *A2M* was correlated with BMI, prostate volume, and age at surgery.

**Supplementary information:**

**Supplementary information** accompanies this paper at 10.1186/s12894-020-00753-9.

## Background

Benign prostatic obstruction (BPO) due to benign prostatic hypertrophy (BPH), enlargement of the prostate gland, is a leading cause of morbidity in men over the age of 40. The prevalence of BPO/BPH increases with advancing age reaching 100% in the ninth decade of life [1]. At present, patients with BPO/BPH are treated with medical therapy consisting of alpha-adrenergic antagonists, 5-alpha-reductase inhibitors or surgery. Medical therapy can lead to considerable side effects including hypotension, erectile dysfunction, gynecomastia, and retrograde ejaculation. Furthermore, surgical treatment can lead to complications such as urinary incontinence, bleeding, urethral stricture disease, or bladder neck contracture [1].

The current guidelines for the management of BPH recommend to treat initially with medical therapy until the disease process progresses to a point at which surgery is a necessity [2]. Unfortunately, BPO/BPH progression is not fully studies [3, 4], and there is a shortage of literature to guide the decisions surrounding when to intervene with definitive surgical therapy in order to avoid adverse outcomes as well as a limited understanding of which patients progress in their disease and why.

Although current BPH and lower urinary tract symptoms (LUTS) studies rarely report racial/ethnic prevalence, it has been reported that men from minority groups, such as Hispanic-Americans (HAs) and African Americans, are at higher risk of developing BPH [5–9]. Additionally, there have been multiple studies reporting the effects of obesity and metabolic syndrome (hypertension, high blood sugar, obesity, waist circumference, and hyperlipidemia) on BPH. Although there is no clear consensus yet, obesity, higher body mass index (BMI), and metabolic diseases were associated risk factors of BPH [7, 9–17]. Despite high prevalence of obesity in HAs and African Americans [18, 19], the relationship between obesity and BPH in these groups and cause for disparities are not fully explored.

It has been hypothesized that pro-inflammatory states due to obesity may be triggers for developing BPH and there may be reactive changes characterized by alterations in the stromal and epithelial microenvironment, reflected in changes in expression of genes involved in inflammatory response and cell growth and development [12, 20]. However, it is still unclear how obesity and metabolic diseases affect BPH pathologic and molecular profiles, especially in medically underserved populations like HAs.

Two main goals of this study were 1) to examine whether there was an association between higher BMI or obesity and prostate volume including HAs who are previously underrepresented in BPH research and 2) to investigate whether expression of three genes, Insulin like growth factor 2 (*IGF2*), alpha-2-macroglobulin (*A2M*), and transforming growth factor beta 3 (*TGFB3*), was correlated with BMI, pre-operative prostate volume, and age at surgery. We also assessed if age and self-identified race/ethnicity modified these relationships.

## Methods

### BPH patients

Electronic medical record of consecutive patients who underwent (HoLEP) surgery for treatment of BPO/BPH between October 2012 and June 2018 were retrospectively reviewed. Patients who had histologically confirmed BPH were included. Patients with (a) pathology diagnoses other than BPH, (b) a history of prostate cancer, or (c) a prior surgical intervention for BPO/BPH were not included. Chart review was conducted on every eligible patient in the identified timeframe. Clinical (e.g., histology, pre-operative prostate volume, and medication) and demographic (e.g., age and race/ethnicity) characteristics as well as BMI, smoking status, and comorbidities (e.g., diabetes, hypertension, and hypolipidemia) were extracted. The pre-operative prostate volume was estimated using the ellipsoid formula (length x width x height x (π/6) based on either transrectal ultrasound or cross-sectional imaging (MRI or CT). The study protocol was approved by the University of Arizona Institutional Review Board.

### Prostate tissue samples

To prepare the surgical specimens for gene expression analysis, hematoxylin and eosin (H&E) slides associated with formalin-fixed paraffin-embedded (FFPE) tissue samples underwent a review by board certified genitourinary pathologists to identify different architectural and cellular components (the stromal-rich and glandular/epithelium-rich areas) on the slides. Then, from the FFPE tissue sample, 1.5 mm^2^ punches from each identified area (the stromal-rich or the glandular/epithelium-rich area) were taken.

### RNA extraction and gene expression analysis

The Roche high Pure FFPET kit was used to extract RNA, and RNA was quantified using Nanodrop. Three genes, *IGF2*, *A2M*, and *TGFB3*, were selected from Luo et al. [21]. Two genes, *A2M* and *TGFB3*, were successfully amplified, but due to technical issues *IGF2* was not successfully amplified. The samples from 30 patients yielded quantifiable RNA expression data using a real time PCR approach. ΔCt method and a housekeeping gene, 18SrRNA, were used for normalization. ΔCt was log2 transformed for statistical analysis.

### Statistical analysis

Chi-square test, independent sample T-test, and Mann–Whitney U-test were performed to characterize eligible patients for this study and patients included for the gene expression study. Linear regression analysis was performed to test the association between BMI and pre-operative prostate volume. Pre-operative prostate volume was log-transformed to normalize. Backward selection approach was used to determine variables to include in the regression mode. Age and race/ethnicity were added to the model to assess if they altered the association between BMI and prostate size. Spearman’s correlation was used to examine whether gene expression was correlated with BMI, prostate volume, and age at surgery. Separate analysis was performed for younger men (age < 70) and older men (age ≥ 70) as well as European Americans (EAs) and HAs to assess if associations were different in these groups.

## Results

**Fig. 1:**
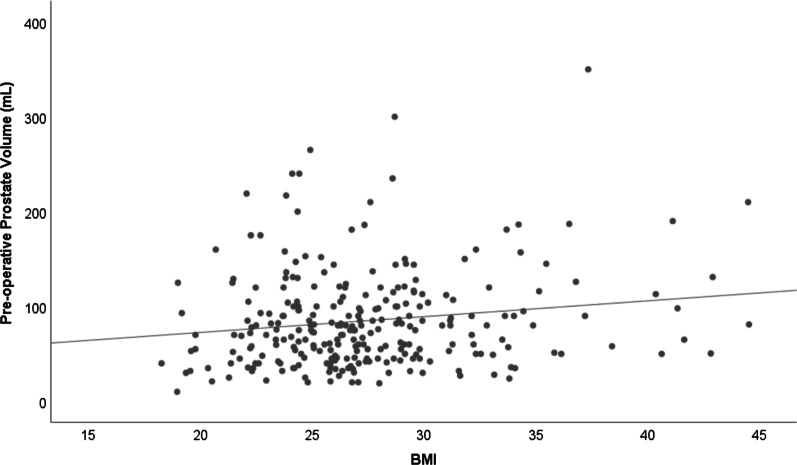
Correlation between pre-operative prostate volume (mL) and BMI (Spearman’s *rho* = 0.123, p = 0.045)

A total of 278 patients who met inclusion criteria were identified. The mean age of patients was 70.6 (SD 8.5). The largest group of patients was EAs (62.9%), and HAs were the second largest group of patients (27.7%). BMI was significantly correlated with pre-operative prostate volume (Spearman’s *rho* = 0.123, *P* = 0.045, Fig. 1). Similar correlations were observed when stratified analysis was performed based on age and race/ethnicity (Additional file 1: Supplementary Table 1). Age at surgery was not correlated with prostate volume but was correlated with BMI (Spearman’s *rho* = −0.169, *P* = 0.005). The correlation was stronger in EAs than in HAs. Median pre-operative prostate volume was 75 mL (Interquartile Range, 48.8–105.0). Characteristics of patients with smaller prostate volume (≤ 75 mL) and larger prostate (> 75 mL) were compared (Table 1). Patients with larger prostates had significantly higher BMI compared to patients with smaller prostate volume (*P* = 0.03). Urinary retention was more common in men with larger prostate (44.7% and 55.9% in men with small and larger prostate respectively, *P* = 0.04).

**Table 1:** BPH patients characteristics stratified by pre-operative prostate volume

	Prostate volume ≤ 75 mL	Prostate volume > 75 mL	*P*
Age, mean (SD)	70.8 (8.8)	70.5 (8.2)	0.81
Race/ethnicity, n (%)			0.48
European Americans	91 (64.5)	84 (61.3)	
Hispanic/latino	35 (24.8)	42 (30.7)	
Other/unknown	15 (10.6)	11 (8.0)	
BMI, median (IQR)	26.3 (24.1–29.0)	27.2 (24.2–29.9)	**0.03**
Smoking history, n (%)			0.30
Never	79 (60.8)	73 (57.5)	
Former smoker	40 (30.8)	48 (37.8)	
Currently smoker	11 (8.5)	6 (4.7)	
Urinary tract Infection, n (%)			0.39
No	113 (80.1)	111 (82.2)	
Yes	28 (19.9)	24 (17.8)	
Urinary retention, n (%)			**0.04**
No	78 (55.3)	60 (44.1)	
Yes	63 (44.7)	76 (55.9)	
Bladder stone, n (%)			0.19
No	128 (90.8)	117 (86.7)	
Yes	13 (9.2)	18 (13.3)	
Diabetes, n (%)			0.23
No	114 (80.9)	104 (76.5)	
Yes	27 (19.1)	32 (23.5)	
Hyperlipidemia, n (%)			0.21
No	80 (56.7)	84 (62.2)	
Yes	61 (43.3)	51 (37.8)	
Hypertension, n (%)			0.30
No	65 (46.1)	57 (42.2)	
Yes	76 (53.9)	78 (57.8)	

*Note*: *P* < 0.05 using Independent sample T-test for age and Chi-square tests for other variables. Bold values indicate statistically significant (*P* < 0.05)

*BMI* body mass index, *BPH* benign prostatic hyperplasia, *IQR* *interquartile range*, *SD* standard deviation

The linear regression analysis was performed to assess if BMI was associated with pre-operative prostate volume (Table 2). BMI, hyperlipidemia, and diabetes remained in the model using the backward selection approach. BMI was positively associated with pre-operative prostate volume (*β* = 0.01, *P* = 0.004). Hyperlipidemia was negatively associated with prostate volume (*β* = −0.08, *P* = 0.02), and a trend for a positive association was observed for diabetes (*β* = 0.07, *P* = 0.099). Additionally, including age at surgery and race/ethnicity in the model did not change the association between BMI and prostate volume. In the race/ethnicity stratified analysis, the association between BMI and prostate volume was similar in both groups, but association was no longer significant in HAs, possibly due to small sample size (Additional file 1: Supplementary Table 2). Interestingly, age at surgery showed a trend for significantly positive association with prostate volume in EAs (*β* = 0.005, *P* = 0.08), but not in HAs. The analysis stratified based on age revealed similar association in younger and older men.

**Table 2:** Linear regression showing association between body mass index and pre-operative prostate volume

	Unadjusted		Adjusted model 1		Adjusted model 2		*P*
	*β*	*P*	*β*	*P*	*β*		
Age at surgery	0.002	0.19			0.003		0.13
BMI	0.009	**0.006**	0.010	**0.004**	0.010		**0.002**
Race/ethnicity							
HAs versus EAs	0.031	0.38			0.030		0.39
Others versus EAs	− 0.043	0.42			− 0.054		0.16
Smoking							
Non-smokers versus former smokers	0.034	0.32					
Non-smokers versus current smoker	− 0.141	**0.03**					
Diabetes	0.051	0.17	0.066	0.099	0.054		0.16
Hyperlipidemia	− 0.047	0.13	− 0.080	**0.02**	− 0.074		**0.02**
Hypertension	0.001	0.98					
UTI	0.008	0.84					
Bladder stone	0.051	0.29					

*Note* Adjusted model 1 is based on backward selection, and BMI, diabetes, and hyperlipidemia were retained in the final mode. Additionally adjusted for age at surgery and race/ethnicity in model 2. Bold values indicate statistically significant (*P* < 0.05)

* BMI* body mass index, *EA* European Americans, *HA* Hispanic Americans, *UTI* urinary tract infection

We then assessed the correlation of gene expression with age at surgery, BMI, and pre-operative prostate volume (Table 3). The characteristics of patients selected for gene expression analysis are presented on Additional file 1: Supplementary Table 3. Expression of the *A2M* gene in the stroma was significantly negatively correlated with age at surgery (*P* = 0.006). In the stratified analysis, the correlation was significant in HAs, men with normal BMI (BMI < 25), and obese men (BMI > 30) (Additional file 1: Supplementary Table 4). *A2M* expression in the gland was significantly positively correlated with prostate volume among older men (Age ≥ 70, *P* = 0.01) and overweight men (BMI 25–30, *P* = 0.04). Among older men, *TGFB3* expression in the gland was significantly positively correlated with BMI (Spearman’s *rho* = 0.709, *P* = 0.007) and showed a trend for significant correlation with prostate volume (*P* = 0.08).

**Table 3:** Correlation between clinical/demographic variables and gene expression (Spearman’s correlation coefficient and *P*-value)

	Age at surgery	BMI	Pre-operative prostate volume
All (n = 30)			
* A2M*			
Gland	– 0.068 (0.72)	0.222 (0.16)	0.277 (0.14)
Stroma	**– 0.498 (0.006)**	0.221 (0.27)	– 0.098 (0.61)
*TGFB3*			
Gland	0.000 (1.00)	0.207 (0.30)	0.326 (0.08)
Stroma	– 0.298 (0.17)	0.321 (0.16)	0.065 (0.77)

*Note*: Bold values indicates statistically significant (P < 0.05)

*BMI* body mass index

## Discussion

In this study, we demonstrated the positive correlation between BMI and pre-operative prostate volume. We also demonstrated gene expression of *TGFB3* and *A2M* and its associations with BMI, preoperative prostate volume, and age at surgery. We also investigated if age and race/ethnicity modified the patterns of correlations.

Although multiple studies have examined the relationships between obesity, particularly abdominal obesity, and BPH risk [7, 12], there are fewer studies that incorporate pre-operative prostate volume, especially in racial/ethnic groups that are affected by both obesity and BPH. Our findings confirmed findings from previous studies and demonstrated a correlation between BMI and prostate volume [9–11, 15–17, 20]. Fowke and colleagues showed that prostate volume was significantly positively associated with BMI, waist-hip ratio, waist circumference, percent body fat, total fat mass, and total lean mass [20]. Kim et al. demonstrated positive correlations between BMI and prostate volume and between BMI and International Prostate Symptom Score among Korean men [11]. A study from Japan even developed a validated model to estimate prostate volume (> 40 mL) based on age, PSA, percent free PSA, and body weight [22]. Despite these studies, there are reports that demonstrate no correlation between BMI or body weight and BPH [23, 24]. Contrary to our finding, Yee et al. investigated the relationship between obesity and LUTS, and they found that men with higher BMI had a smaller prostate than men with lower BMI at baseline [25]. Moreover, weight loss did not improve LUTS after intervention.

Previous studies also demonstrated that Hispanic ethnicity was associated with BPH risk with increased rates of reporting LUTS [7, 8]. Obesity is more prevalent among HAs than EAs [26, 27]. The 2014 National Health Interview Survey analysis results also show that HAs have a higher prevalence of diabetes than EAs [28]. Moreover, the prevalence of diabetes is slightly higher in Mexican Americans, especially Mexican American men, than HAs of other origins [26]. We hypothesized that men from minority groups, such as HAs, who were obese and had metabolic diseases were disproportionately affected by BPH. When stratifying by race/ethnicity, both EA and HA had a positive correlation between BMI and preoperative prostate volume, but correlation was significant only for EAs, probably due to small sample size for HAs.

Contrary to our hypothesis, hyperlipidemia was negatively associated with pre-operative prostate volume, and we did not observe significant association for diabetes or hypertension. There have been multiple studies interested in demonstrating the link between metabolic syndrome and BPH, but findings have been inconsistent. Some studies have reported how diabetes [13, 14] and hyperlipidemia [13, 29] were correlated with BPH, while other studies have failed to demonstrate an association [23, 24]. Egan and colleagues investigated the correlation between metabolic diseases and BPH in National Health and Nutrition Examination Surveys [24]. They found that cardiovascular disease, diabetes, or hypertension were not associated with BPH after adjusting for social and behavioral factors. When breaking down the components of metabolic syndrome or looking at metabolic syndrome as a whole, Telli et al. found no evidence to support any association between metabolic syndrome and LUTS [23]. The clinical significance of current and previous studies is uncertain, because of the consistent, but weak association between obesity and prostate volume, and inconsistent associations between metabolic diseases and BPH. Since obesity rates continue to increase in racial/ethnic minority groups, impact of obesity on BPH and its clinical implications need further investigation. The obesity-driven BPH may have a different pathophysiological basis from the hormonally-driven or age related progression of BPH, and understanding of pathophysiological basis of obesity-driven may open up a new avenue for treatment.

To explore the biological basis of obesity-driven BPH, we examined the correlations between obesity and expression of genes that are potentially related to obesity. We demonstrated how expression of two genes, *A2M* and *TGFB3*, were correlated with age at surgery, BMI, or preoperative prostate volume. These two genes along with *IGF2* were selected as genes of interest in BPH because they were over-expressed in BPH compared to normal prostate tissue in a previous study by Luo et al. [21] and these gene may play roles in obesity [30–33]. *A2M* was also over-expressed in BPH compared to normal prostate tissue in another study [34]. *TGFB3* and *IGF2* are both growth factors, and *TGFB3* encodes a ligand of the transforming growth factor-beta (TGF-beta) superfamily. *A2M* encodes a protease inhibitor and cytokine transporter, and the protein encoded by *A2M* can inhibit inflammatory cytokines and interrupt inflammatory cascades. A more recent molecular profiling study using RNA sequencing further demonstrated that *A2M* and *TGFB3* were over-expressed in BPH [35]. However, this study identified *BMP5* and *CXCL13* as the two most significantly over-expressed genes in BPH. *BMP5* (bone morphogenetic protein 5) also encodes a ligand of the TGF-beta superfamily. Ligands of this protein family bind TGF-beta receptors and activate SMAD family transcription factors. Dysregulation of *TGFB3* and *BMP5* may affect expression of downstream genes that are involved in BPH progression. *CXCL13* (C-X-C motif chemokine ligand 13) is an antimicrobial peptide and CXC chemokine, a small cytokine. Both growth factors and immune response are likely to be involved in epithelial and stromal growth in the prostate promoting development and progression of BPH [4, 36–38], but growth factors and inflammatory response gene may not influence prostate volume uniformly in all racial/ethnic and age groups. Moreover, a molecular profiling study identified two distinct subtypes of BPH, subtype A enriched with stromal signatures and subtype B associated with obesity and hypertension [39]. This current study also observed different gene expression correlation patterns for different cell types, race/ethnicity, and age groups. The difference in gene expression patterns between cell types or between patients’ demographic backgrounds and co-morbid conditions needs to be further investigated with a larger sample.

The limitations of this study include its retrospective nature and small sample size at a single institution. Only 278 patients met our inclusion criteria, but we provide surgical care for a diverse patient population due to our location in the Southwestern United States. To our knowledge, this is the first study to explore the relationship between obesity and BPH in HA men in the United States. Secondly, the prostate volume was measured using ultrasound, CT, or MR. Different measurement methods may create systematic bias causing false positive or negative associations. Moreover, we explored the correlation between expression of only three genes and BPH characteristics in a small subset of patients, and we failed to amplify one of the genes, *IGF2*. All the patients included were had advanced BPH that required a surgical treatment and they were receiving medical management with alpha blockade and/or 5-alpha reductase inhibitors. Medications may have altered the correction between BMI and prostate volumes or between metabolic diseases and prostate volumes. Inclusion of patients with less severe BPH or BPO who have not undergone surgical or therapeutic treatment and a multi-institutional study with a larger sample size and a more diverse patient population is warranted to further investigate the impact of obesity or obesity related diseases on BPH and BPO characteristics. Finally, there is a paucity of data regarding molecular risk factors in patients with BPH. Further studies are needed to identify genes linked to BPH, so precision medicine can be used to predict disease progression, treatment response, and clinical outcomes.

## Conclusions

We demonstrated the positive association between BMI and pre-operative prostate volume. We also demonstrated the association between gene expression of *TGFB3* and *A2M* with BMI, preoperative prostate volume and age at surgery. Further studies are needed to confirm these findings.

## Supplementary information

**Additional file 1**. Suppmentary Tables.

## Data Availability

All the data are available upon request.

## Abbreviations

BMI: Body mass index
BPH: Benign prostatic hyperplasia
BPO: Benign prostatic obstruction
CT: Computed tomography
EA: European Americans
FFPE: Formalin-fixed paraffin-embedded
HA: Hispanic Americans
HoLEP: Holmium enucleation of the prostate
LUTS: Lower urinary tract symptoms
MRI: Magnetic resonance imaging
PCR: Polymerase chain reaction
RNA: Ribonucleic acid
SD: Standard deviation
