# Supplementary material for: Correlation between body mass index and prostate volume in benign prostatic hyperplasia patients undergoing holmium enucleation of the prostate surgery
Source: BMC Urol. 2021 Jun 10;21:88. doi: 10.1186/s12894-020-00753-9 (PMC8191122; doi:10.1186/s12894-020-00753-9)
Supplement: Supplementary file 1 — Additional file 1. Suppmentary Tables. [file 12894_2020_753_MOESM1_ESM.docx]

**Supplementary Tables**

**Supplementary Table 1** Spearman’s correlation (*rho* and *P*-value) among three factors (age, BMI and pre-op prostate volume) in all samples and stratified by age (<70 vs. ≥70) or race/ethnicity

|  | BMI and Prostate Volume | Age at Surgery and Prostate Volume | Age at Surgery and BMI |
| --- | --- | --- | --- |
| All (n=268) | 0.123 (**0.045**) | 0.028 (0.64) | -0.169 (**0.005**) |
| Age |  |  |  |
| <70 (n=123) | 0.111 (0.22) |  |  |
| ≥70 (n=145) | 0.141 (0.09) |  |  |
| Race/Ethnicity |  |  |  |
| European Americans (n=169) | 0.153 (**0.047**) | 0.063 (0.41) | -0.219 (**0.004**) |
| Hispanic Americans (n=73) | 0.129 (0.28) | 0.009 (0.94) | -0.182 (0.12) |
| Unknown/others (n=26) | -0.116 (0.57) | 0.051 (0.80) | 0.191 (0.35) |

Note: Bold values indicates statistically significant (*P*<0.05)

**Supplementary Table 2** Association between BMI and pre-operative prostate volume stratified by age (<70 vs. ≥70) or race/ethnicity

|  | Age<70 | | Age≥70 | | Non-Hispanic Whites | | Hispanic Americans | |
| --- | --- | --- | --- | --- | --- | --- | --- | --- |
|  | *β* | *P* | *β* | *P* | *β* | *P* | *β* | *P* |
| Age at Surgery |  |  |  |  | 0.005 | 0.08 | 0.002 | 0.59 |
| Race/ethnicity |  |  |  |  |  |  |  |  |
| HAs vs. EAs | 0.041 | 0.43 | 0.016 | 0.74 |  |  |  |  |
| Others vs. EAs | -0.056 | 0.49 | -0.071 | 0.33 |  |  |  |  |
| BMI | 0.009 | 0.09 | 0.010 | **0.02** | 0.013 | **0.003** | 0.012 | 0.08 |
| Diabetes | 0.081 | 0.22 | 0.047 | 0.33 | 0.046 | 0.38 | 0.029 | 0.66 |
| Hyperlipidemia | -0.076 | 0.13 | -0.069 | 0.11 | -0.077 | 0.065 | -0.106 | 0.09 |

Note: Bold values indicates statistically significant (*P*<0.05)

Abbreviations: BMI, body mass index; EA, European Americans; HA, Hispanic Americans

**Supplementary Table 3** Characteristics of patients (n=30) included in gene expression analysis

| Age, mean (SD) | 69.1 (10.7) |
| --- | --- |
| Race/ethnicity, n (%) |  |
| European Americans | 17 (56.7%) |
| Hispanic Americans | 11 (36.7%) |
| Others | 2 (6.7%) |
| BMI, mean (SD) | 27.3 (5.6) |
| Pre-Operative Prostate Volume, median (IQR) | 52.6 (35.0-90.0) |

Abbreviations: BMI, body mass index; IQR, interquartile range; SD, standard deviation

**Supplementary Table 4** Correlation between clinical/demographic variables and gene expression stratified by age, BMI, or race/ethnicity (Spearman’s correlation coefficient and *P*-value)

|  | Age at Surgery | BMI | Pre-Operative Prostate Volume |
| --- | --- | --- | --- |
| *European Americans (n=17)* |  |  |  |
| *A2M* |  |  |  |
| Gland | -0.041 (0.88) | 0.394 (0.13) | 0.337 (0.19) |
| Stroma | -0.238 (0.38) | 0.321 (0.24) | -0.200 (0.46) |
| *TGFB3* |  |  |  |
| Gland | -0.047 (0.86) | 0.339 (0.22) | 0.377 (0.15) |
| Stroma | 0.047 (0.87) | 0.341 (0.26) | 0.339 (0.24) |
| *Hispanic Americans (n=10)* |  |  |  |
| *A2M* |  |  |  |
| Gland | -0.127 (0.71) | -0.164 (0.65) | 0.182 (0.59) |
| Stroma | **-0.736 (0.01)** | -0.115 (0.75) | 0.009 (0.98) |
| *TGFB3* |  |  |  |
| Gland | -0.309 (0.36) | 0.139 (0.70) | 0.336 (0.31) |
| Stroma | **-0.810 (0.02)** | 0.571 (0.18) | 0.190 (0.65) |
| *Age <70 (n=16)* |  |  |  |
| *A2M* |  |  |  |
| Gland |  | -0.054 (0.85) | -0.071 (0.80) |
| Stroma |  | 0.304 (0.27) | -0.05 (0.85) |
| *TGFB3* |  |  |  |
| Gland |  | -0.327 (0.25) | -0.039 (0.89) |
| Stroma |  | 0.255 (0.45) | -0.007 (0.98) |
| *Age 70 or older (n=14)* |  |  |  |
| *A2M* |  |  |  |
| Gland |  | 0.456 (0.12) | **0.648 (0.01)** |
| Stroma |  | 0.063 (0.85) | 0.132 (0.67) |
| *TGFB3* |  |  |  |
| Gland |  | **0.709 (0.007)** | 0.486 (0.08) |
| Stroma |  | 0.467 (0.17) | 0.045 (0.89) |
| *BMI <25 (n=13)* |  |  |  |
| *A2M* |  |  |  |
| Gland | -0.429 (0.14) |  | 0.196 (0.52) |
| Stroma | **-0.685 (0.01)** |  | -0.095 (0.77) |
| *TGFB3* |  |  |  |
| Gland | -0.407 (0.17) |  | -0.19 (0.53) |
| Stroma | -0.545 (0.08) |  | 0.005 (0.99) |
| *BMI 25-30 (n=8)* |  |  |  |
| *A2M* |  |  |  |
| Gland | 0.262 (0.53) |  | **0.738 (0.04)** |
| Stroma | 0.190 (0.65) |  | -0.143 (0.74) |
| *TGFB3* |  |  |  |
| Gland | 0.643 (0.09) |  | 0.548 (0.16) |
| Stroma | 0.300 (0.62) |  | -0.600 (0.29) |
| *BMI >30 (n=7)* |  |  |  |
| *A2M* |  |  |  |
| Gland | 0.436 (0.33) |  | -0.286 (0.54) |
| Stroma | **-0.764 (0.046)** |  | -0.429 (0.34) |
| *TGFB3* |  |  |  |
| Gland | 0.493 (0.32) |  | -0.200 (0.70) |
| Stroma | -0.308 (0.61) |  | 0.500 (0.39) |

Note: Bold values indicates statistically significant (*P*<0.05)

Abbreviations: BMI, body mass index
